# Interfacial Structure and Reactions in Li_6.7_Al_0.3_La_3_Zr_2_O_12_-Doped Polycarbonate-Based Composite Polymer Electrolytes

**DOI:** 10.1021/acsapm.4c03865

**Published:** 2025-02-28

**Authors:** Kenza Elbouazzaoui, Edvin K.W. Andersson, Yi-Chen Weng, Daniel Friesen, Kristina Edström, Erika Giangrisostomi, Ruslan Ovsyannikov, Daniel Brandell, Jonas Mindemark, Maria Hahlin

**Affiliations:** † Department of Chemistry−Ångström Laboratory, Uppsala University, Box 538, SE-751 21 Uppsala, Sweden; ‡ Department of Physics and Astronomy, Uppsala University, Box 516, 751 05Uppsala, Sweden; § Institute for Methods and Instrumentation for Synchrotron Radiation Research, Helmholtz-Zentrum Berlin für Materialien und Energie, Albert-Einstein-Str. 15, 12489 Berlin, Germany

**Keywords:** composite polymer electrolyte, PTMC, LLZO, interface, photoelectron spectroscopy

## Abstract

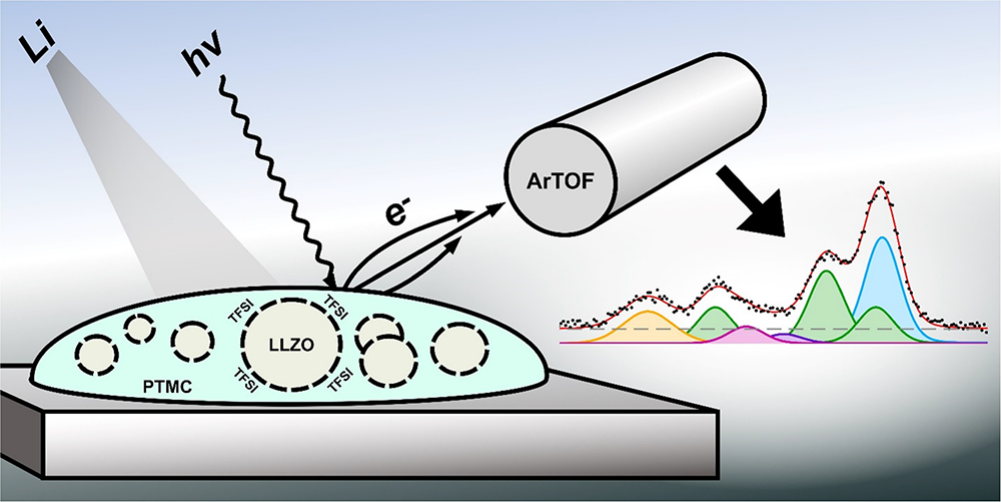

Solid composite polymer electrolytes (CPEs) are complex mixtures of ceramics, polymers, and lithium salts, where the interfaces between the different phases play an important role for stability, conductivity, and compatibility with electrode materials. In this study, two interfacial phenomena of CPEs consisting of lithium lanthanum zirconium oxide (LLZO) ceramic fillers in poly(trimethylene carbonate) (PTMC) with lithium bis(trifluoromethanesulfonyl)imide (LiTFSI) salt are studied. First, the LLZO-polymer electrolyte interfaces are investigated. Second, the stability of this CPE material vs a Li-metal electrode is explored, by employing soft X-ray photoelectron spectroscopy (PES) in combination with in situ deposition of Li. Three different LLZO loadings in PTMC are investigated: 30, 50, and 70 wt %. The concentration of LiTFSI follows that of the particle concentration at the surface of the samples, where the CPE with 50 wt % bulk content of LLZO exhibits the highest surface concentrations of both salt and ceramic. This shows an affinity for the salt at the LLZO surface. Furthermore, the stability of the CPEs against Li is studied after in situ Li deposition and shows that PTMC can decompose, potentially forming polypropylene at the CPE|Li interface, with the CPE at 50 wt % of LLZO showing the most pronounced PTMC and TFSI breakdown. This is in agreement with the observed properties for the polymer-ceramic interfaces and highlights the decisive role of LiTFSI accumulation on the surface of the ceramic particles, both for ionic transport and chemical stability.

## Introduction

Composite polymer electrolytes (CPEs) constitute a class of Li-battery solid electrolytes that ideally combines the benefits of polymers and ceramics into a single material with enhanced ionic conductivity, high Li^+^ transference number, widened electrochemical stability, mechanical flexibility, and low interfacial resistance with the electrodes.^1^ At the same time, it has been shown that the overall properties of CPEs are dependent on many factors, e.g., polymer crystallinity, salt concentration, ceramic particle type and size, and the interfaces between the components, making it difficult to achieve these desired properties.^2−5^ One commonly used ceramic filler for CPEs is garnet-type Li_7_La_3_Zr_2_O_12_ (LLZO), motivated by its high intrinsic ionic conductivity and wide electrochemical stability. While LLZO electrolytes suffer from brittleness and poor interfacial contacts, particularly during cycling due to volume changes in the electrodes, the addition of a flexible polymer electrolyte matrix surrounding the conducting particles can mitigate this.^3,6−8^

In this context, LLZO has been widely used with poly(ethylene oxide) (PEO) to fabricate CPEs,^9−12^ and it has been observed that the ionic conductivity in PEO:LLZO CPEs is highly dependent on the LLZO ceramic concentration.^2^ Moreover, it has also been seen that the potential benefits of LLZO are often limited due to that its surface chemistry is governed by the presence of Li_2_CO_3_,^13−15^ resulting in higher resistivity. A Li_2_CO_3_-covered LLZO ceramic filler can also influence the interfacial chemistry between the CPE and the battery electrodes, i.e., the cycling stability vs Li metal.^16−20^

While PEO is the most investigated solid polymer electrolyte (SPE) host material for LLZO-based CPEs, it is a semicrystalline polymer, making it difficult to clearly elucidate different transport phenomena in the electrolyte, since the addition of particles will also influence the crystalline content. In contrast, poly(trimethylene carbonate) (PTMC) is a fully amorphous SPE host,^21−24^ albeit with lower ionic conductivity than PEO.^25^ With PTMC as the polymer matrix in CPEs, any effects of changes in crystallinity associated with introducing ceramic fillers into a semicrystalline polymer matrix are avoided, and the intrinsic effect of the filler on the ion transport properties can thus be better isolated. In previous studies, we have shown that the addition of LLZO can improve the overall ionic conductivity of PTMC-based SPEs at low-to-moderate loadings, but that higher LLZO ceramic loadings (>40 wt %) lead to a detrimental effect on conductivity.^13^ Moreover, the presence of Li_2_CO_3_ on the surface of the LLZO particles appears to be a decisive factor in this sense, resulting in lower ionic conductivity with high LLZO loadings in CPEs.^14,26,27^ Therefore, it is crucial to study the interfacial chemistry in these CPE materials in greater detail.

Furthermore, the interfacial stability with the Li-metal electrode is another factor controlling electrolyte performance. In previous studies, we have explored the surface properties of SPEs based on different polymers and salts, and the results showed that PTMC:LiTFSI exhibits the most severe decomposition compared to other LiTFSI-based systems with poly(ε-caprolactone) (PCL) or PEO, but appears to render an interphase that is more stable over time.^28^ In this study, we investigate how this process is affected by the introduction of LLZO particles into the solid electrolyte.

Soft X-ray photoelectron spectroscopy (PES) combined with in situ Li deposition can be employed to gain more insights into the surface properties of solid-state electrolytes and their functionality with Li metal. The technique consists of irradiating the sample by soft X-rays while varying the photon energy,^29^ to enable depth-sensitive surface measurements.^30^ In addition, the LowDose beamline used offers the possibility to perform measurements with low intensity, thus reducing the risk of radiation damage. While this technique on its own is useful to study the LLZO/SPE interfacial properties, it can also be followed by in situ deposition of lithium metal on top of the CPE surface under ultrahigh vacuum (UHV) conditions. This allows the formation of an interphase between Li metal and the CPE, thereby overcoming experimental difficulties related to ex-situ XPS analysis on polymer/composite electrolyte-electrode interfaces.^28,30,31^

In this work, soft X-ray PES combined with in situ Li deposition is employed to investigate the intrinsic polymer-ceramic interfaces in CPEs and interfaces formed when in contact with Li metal, based on a PTMC:LiTFSI polymer matrix with LLZO ceramic filler at three different loadings (30, 50, and 70 wt %). The study aims to provide both insights into the effect of LLZO surface chemistry on the surface properties of PTMC:LLZO CPEs themselves and how the material changes after Li deposition. While the results show that the addition of LLZO particles into the SPE does not contribute to significantly less electrolyte decomposition when in contact with Li metal, but rather to the formation of somewhat different decomposition products, the clear effect is instead that of LiTFSI accumulation on the LLZO particles, which in turn are partially covered by Li_2_CO_3_. This appears to play an important role in the transport properties of the CPEs.

## Experimental Section

### Materials

Trimethylene carbonate (TMC; Richman Chemicals) and stannous 2-ethylhexanoate (95%; Sigma-Aldrich) were handled inside an Ar-filled glovebox. Li_2_CO_3_ (99.99%), La(OH)_3_ (99.99%), ZrO_2_ (99%), and isopropanol (>99.8%), from Sigma-Aldrich, and Al_2_O_3_ (99.9%; VWR) were used as received for LLZO synthesis. Prior to CPE preparation, lithium bis(trifluoromethylsulfonyl)imide (LiTFSI; BASF) was dried at 120 °C under vacuum in a Buchi oven for 48 h, while anhydrous acetonitrile (99.8%; Sigma-Aldrich) was used as received.

### Synthesis of Poly(trimethylene carbonate) (PTMC)

PTMC was synthesized via a bulk ring-opening polymerization as previously described.^22^ In brief, to 0.2 mol of trimethylene carbonate (TMC) was added 0.04 mmol of a solution of tin(II) 2-ethylhexanoate (Sn(Oct)_2_) in dry toluene in a stainless-steel reactor, which was kept in an oven at 130 °C for 72 h. Once the polymerization was complete, the polymer was obtained as a transparent and rubbery solid, with an approximate molecular weight of Mn of 380,000–400,000 g mol^–1^, as determined by gel permeation chromatography (GPC).

### Synthesis of Li_6.7_Al_0.3_La_3_Zr_2_O_12_ (LLZO)

LLZO was synthesized following a typical solid-state synthesis method as described previously.^13^ Briefly, Li, La, Zr, and Al reagents were mixed together using a planetary ball-miller before being heat-treated at 1000 °C for 12 h in air with a ramp of 2 °C/min. The obtained white powder was ball-milled and stored immediately inside an Ar-filled glovebox before use. The particle size was 3–5 μm, according to our previous paper.^13^

### PTMC:LLZO Composite Electrolyte Preparation

**Figure 1:**
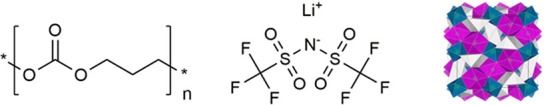
Structure of PTMC, LiTFSI, and a LLZO unit cell, from left to right. The LLZO unit cell is represented by ZrO_6_ octahedra (in blue) and LaO_8_ dodecahedra (in violet).

CPEs were prepared via a two-step process. First, both PTMC and LiTFSI (28 wt %) were dissolved in acetonitrile ([Polymer]/[Solvent] = 0.1 g/mL) at 60 °C for 12 h. The obtained solution was relatively viscous for better dispersion of LLZO particles. LLZO powder was added to the polymer solution and ball-milled at 25 Hz for 15 min under an Ar atmosphere. The obtained slurries were transferred to PTFE molds prior to vacuum drying. 30, 50, and 70 wt % of LLZO particles were chosen, and the resulting CPEs were named CPE30, CPE50, and CPE70, respectively. The structure of the three components of CPEs (polymer, salt, and ceramic) is displayed in Figure 1.

### Photoelectron Spectroscopy and In Situ Li Deposition

Photoelectron spectroscopy (PES) measurements were conducted at the LowDosePES end station on the PM4 beamline of the BESSY II electron storage ring, managed by the Helmholtz-Zentrum Berlin für Materialien and Energie.^29^ The station is equipped with a high-transmission angular-resolved time-of-flight (ArTOF) spectrometer, featuring a ±30° acceptance angle. The setup is optimized for analyzing radiation-sensitive samples such as solid polymer electrolytes. Throughout the measurement, the analysis chamber’s pressure was constantly maintained at or below the low 1 × 10^–9^ mbar range, typically reaching levels around 1 × 10^–10^ mbar.

The core levels measured were S 2p, C 1s, N 1s, O 1s, and F 1s. Zr and La were not measured due to time constraints and a low signal at the pristine surface, as previously reported.^13,32^ To get a consistent depth of analysis, the photoelectron kinetic energy was kept between 300 and 310 eV. The photon energies were therefore set at 475, 600, 710, 845, and 1000 eV. The C 1s core level was recorded for each photon energy every time the photon energy was changed and used for internal binding energy calibration. Specifically, the position of the TFSI carbon peak in the C 1s spectra was set to 293.0 eV in binding energy, and the related core-level spectra were then adjusted accordingly. The measurement on each core level was done on a fresh sample spot when changing the photon energy. Each respective core level was measured with the same number of sweeps and presented as measured. All core levels were measured before and after a 15 min Li deposition step, achieved using a resistively heated Li dispenser (S.A.E.S group) at 10.0 A and 3.5–3.6 V (a method developed by Wenzel et al.).^33^ During Li deposition, the pressure in the deposition chamber was in the range of 10^–8^ mbar. All PES data treatment (energy calibration, curve fitting) of synchrotron PES data was done using Igor pro version 9.0.1.2 using the Spectral Analysis by Curve Fitting (SPANCF) package by Edwin Kukk.^34^ In this package, PES data fitting was performed using a pseudo-Voight equation as the peak shape for all peaks in the curve fit, and the optimization algorithm used was the simplex algorithm.

## Results and Discussion

Three different CPEs with increasing LLZO ceramic loadings (30, 50, and 70 wt %) were investigated: CPE30, CPE50, and CPE70. Through PES combined with in situ Li deposition, the surface of the CPEs was first characterized in the pristine state, followed by a Li deposition step, allowing the formation of a CPE|Li interface, after which this new surface/interface was characterized. Below, the PES spectra with the curve fit and their interpretation are presented for each core level. Later, all PES data are correlated and further discussed.

### PES Results and Assignments

**Figure 2:**
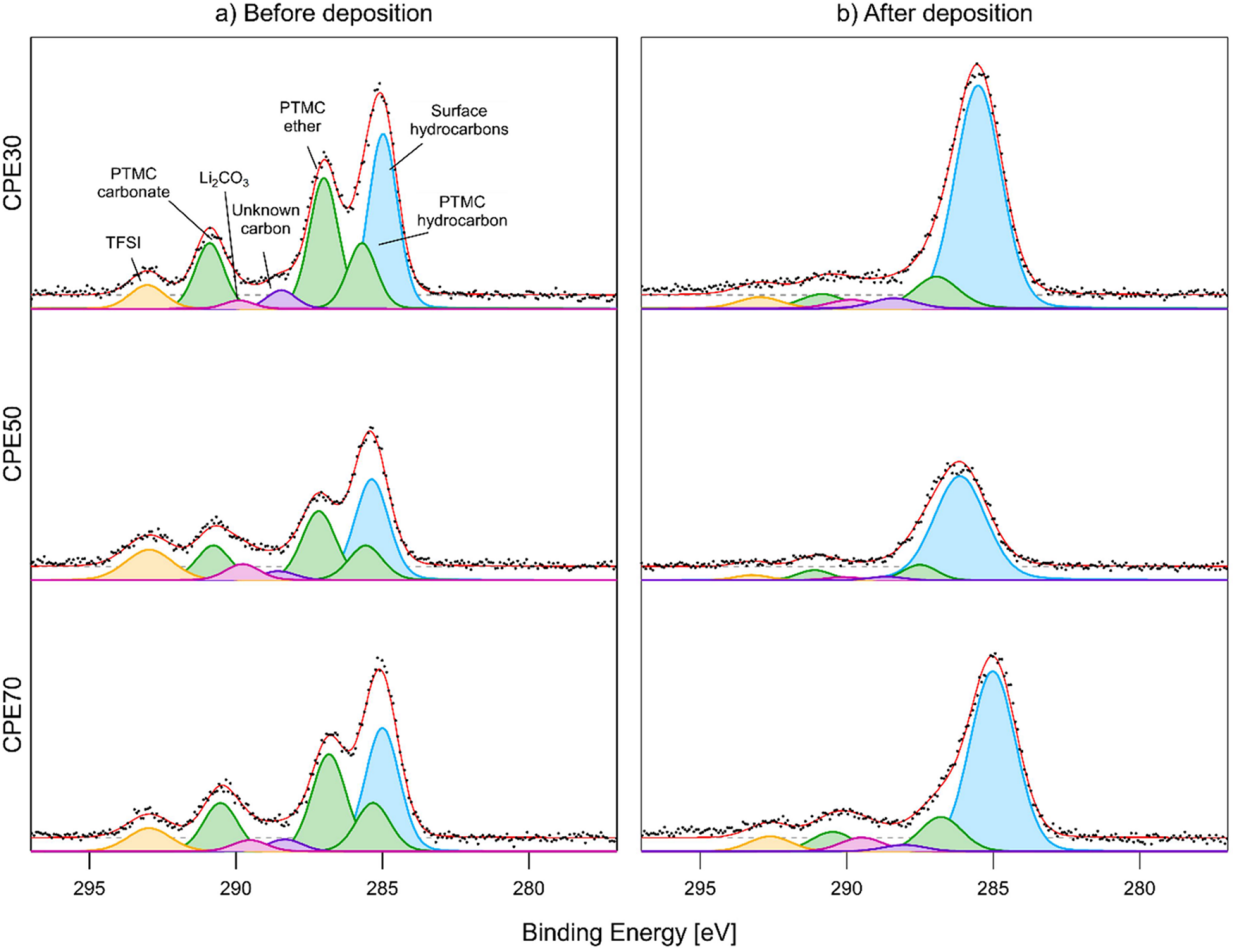
C 1s spectra of CPE30, CPE50, and CPE70, in descending order. To the left (a) is before Li deposition, and to the right (b) is after deposition.

**Figure 3:**
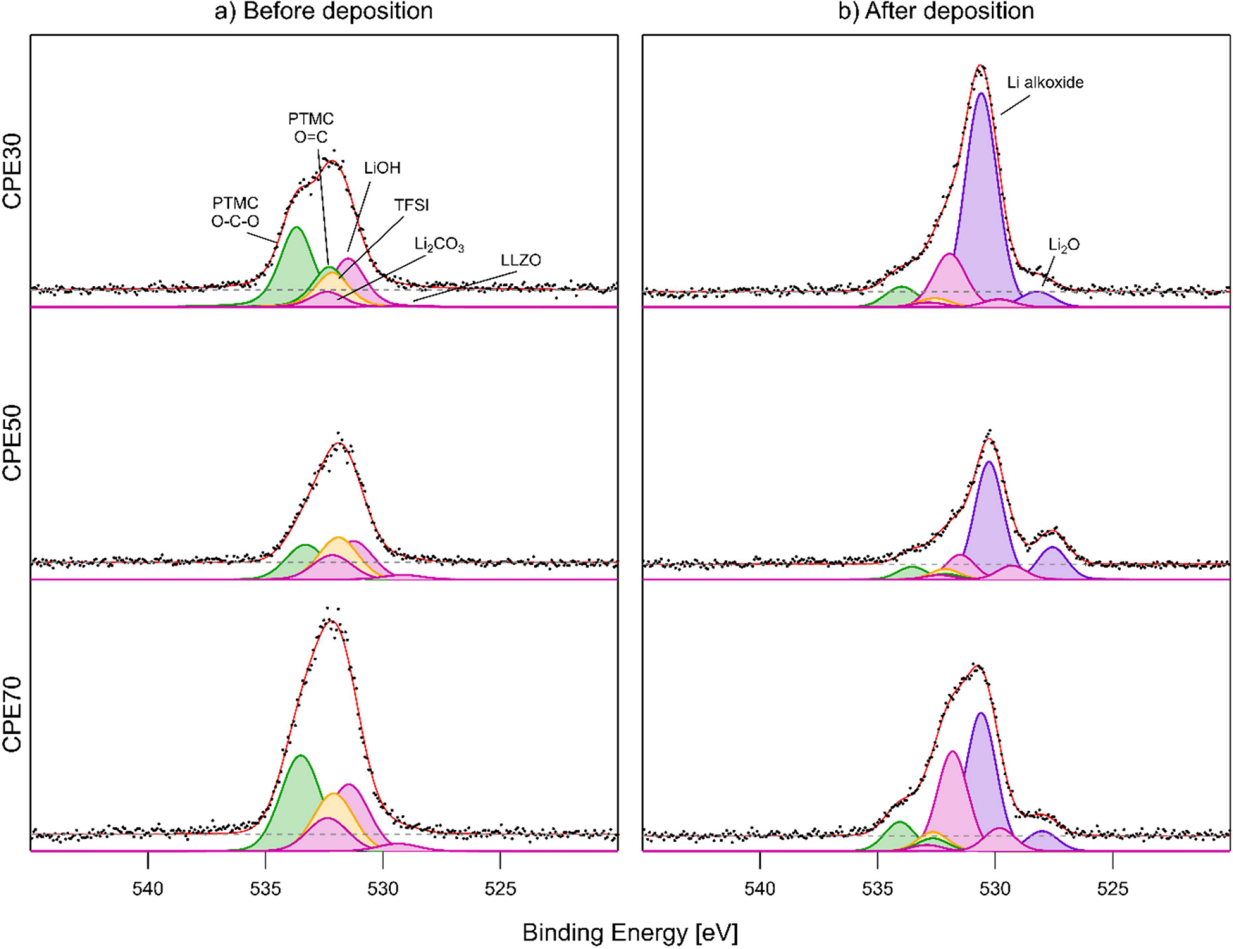
O 1s spectra of CPE30, CPE50, and CPE70, in descending order. To the left (a) is before deposition, and to the right (b) is after deposition.

**Figure 4:**
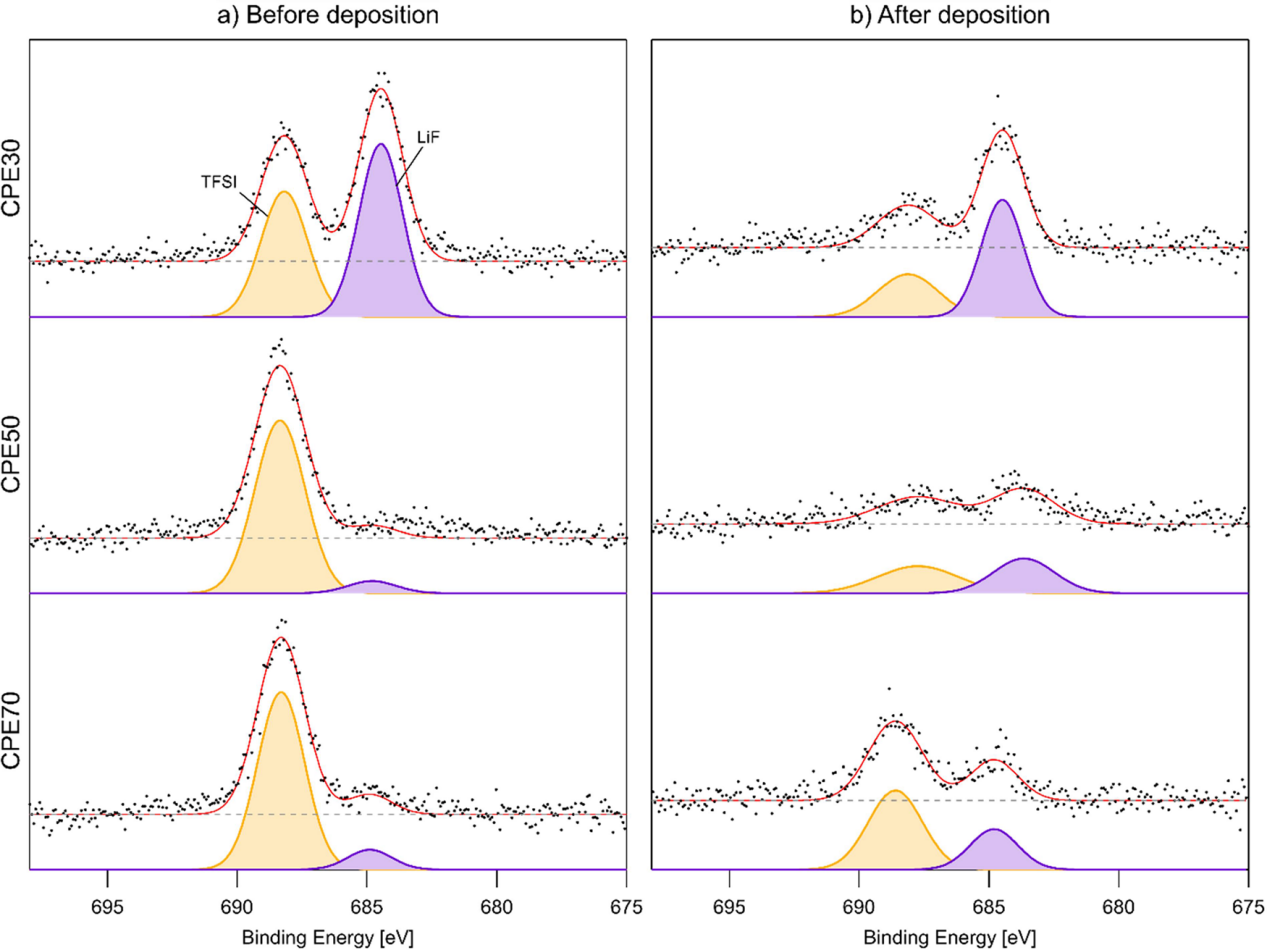
F 1s spectra of CPE30, CPE50, and CPE70, in descending order. To the left (a) is before deposition, and to the right (b) is after deposition.

The C 1s, O 1s, and F 1s PES spectra, before and after the deposition of lithium onto the CPE film surface, are shown in Figures 2–4, and the obtained areas for the curve fits for the C 1s and O 1s peaks are shown in Tables 1 and 2, respectively. The curve fits for the PES spectra are challenging, considering that the samples contain several compounds with contributions that overlap in binding energy. However, by restraining the fit based on information from previous references and utilizing information from clearly separate peaks in constraints for another core level, the relative abundance of many of the most important compounds in these CPEs can be obtained. In brief, the intensity ratios of the polymer peaks for the C 1s and O 1s fits were locked to the stoichiometric values, and the binding energy (BE) difference between the peaks was set to be the same as previous reference values.^35^ Then, starting with the pristine C 1s spectra, a clear relative intensity ratio between LiTFSI and the PTMC C–O oxygen peak could be obtained. As no other compounds contribute to the spectra in these binding energy ranges, this LiTFSI-to-PTMC ratio should, by default, be the same for the relative intensity of LiTFSI and PTMC in the O 1s spectra and thus applied as a constraint to the O 1s fit. Moreover, the Li_2_CO_3_—which is expected to exist on the LLZO surface—is expected to appear at a slightly lower binding energy than the carbonyl oxygen from PTMC in the O 1s spectra due to the difference in electron density around the carbonate oxygen. These considerations are essential in order to separate the many overlapping peaks in this O 1s binding energy range. The LLZO 1s peak, on the other hand, is clearly separate from the other contributions. Generally, the presented trends drawn from the curve fits have been based on the relative peak areas within each core-level spectrum.

**Table 1:** Area of Peaks in Figure 2 Calculated from the Peak Intensities as a Ratio of the Total Peak Areas^a^

	*CPE30*		*CPE50*		*CPE70*	
minutes of lithium deposition	0	15	0	15	0	15
total hydrocarbon intensity	0.489	0.738	0.443	0.822	0.464	0.688
total carbonate intensity	0.149	0.079	0.165	0.062	0.161	0.116
PTMC ether peak intensity	0.266	0.108	0.225	0.073	0.262	0.120
LiTFSI peak intensity	0.057	0.038	0.135	0.024	0.079	0.052
unknown carbon peak intensity	0.037	0.035	0.029	0.016	0.032	0.022

a Total carbonate includes C=O from PTMC and Li_2_CO_3_.

**Table 2:** Area of Peaks in Figure 3 Calculated from the Peak Intensities as a Ratio of the Total Peak Areas

	*CPE30*		*CPE50*		*CPE70*	
minutes of lithium deposition	0	15	0	15	0	15
PTMC C=O intensity	0.365	0.060	0.216	0.057	0.309	0.084
PTMC C–O intensity	0.183	0.025	0.108	0.026	0.155	0.037
LiTFSI peak intensity	0.157	0.026	0.259	0.047	0.187	0.055
Li_2_CO_3_ peak intensity	0.069	0.014	0.151	0.020	0.107	0.018
LiOH peak intensity	0.221	0.160	0.238	0.112	0.217	0.286
LLZO peak intensity	0.005	0.023	0.028	0.063	0.024	0.066
Li alkoxide intensity	N/A	0.646	N/A	0.581	N/A	0.397
Li_2_O peak intensity	N/A	0.046	N/A	0.146	N/A	0.058

Based on this fitting model, in the C 1s spectra before Li deposition (Figure 2a), all CPEs display the three expected peaks from PTMC, the peak expected from LiTFSI, as well as surface hydrocarbons, as in our previous study of the PTMC:LiTFSI system.^28^ However, unlike our previous study, a peak appears between the carbonate peaks and the ether peak, which remains unidentified. After the deposition of lithium metal, the same trend as previously observed for PTMC:LiTFSI is noted, i.e., a substantial increase in hydrocarbons, which here is the most pronounced for CPE50. In general, based on the relative change in peak areas of most peaks, we find that both polymer and salt decomposition are most pronounced for CPE50, then CPE70, followed by CPE30.

In the O 1s spectra measured before deposition (Figure 3a), the following peaks were used to fit the spectra: C–O, C=O, LiTFSI oxygen, LLZO, Li_2_CO_3_, and LiOH, where the first two originated from the polymer and the latter three originated from species on the LLZO surface.^36^ After lithium deposition, Li alkoxide and Li_2_O are formed on the surface, which is in line with previous observations.^28^ Furthermore, the LiOH peak becomes more dominant in the spectra for all three samples after deposition. When comparing the three different samples, Li alkoxide is most pronounced for CPE70, while the Li_2_O peak is most pronounced for CPE50, and thus forms to the largest extent in those samples. Although the LLZO peak overlaps with the Li alkoxide peak (making an unambiguous curve fit challenging), another notable trend in the curve fit is that the LLZO peak increases for all samples after Li deposition, showing that after the lithium deposition step, the LLZO is more exposed. Most of these observed changes are further discussed below.

For the F 1s core level, the peak expected from LiTFSI shows up at ∼688 eV, and small amounts of LiF, possibly caused by beam damage, are also visible before deposition for the CPE70 and CPE50 samples. The reason behind the relatively large amount of LiF on the CPE30 sample before Li deposition is, however, unclear. Regardless, after deposition, LiF is formed for all CPEs, similar to what has been observed for PTMC:LiTFSI without LLZO.^28,30^ The relative amount of LiF forming after Li deposition is largest for CPE50, followed by CPE70 and finally CPE30, which matches the order of descending degree of decomposition seen from the C 1s spectra. Furthermore, the S 2p and N 1s spectra (Figure S1) confirm the decomposition of LiTFSI into Li_2_S and what has previously been attributed to Li_3_N.^28^ In the S 2p spectrum, there is a peak that previously has been attributed to an unknown Li_*x*_S_*y*_O_*z*_ compound. Wu et al. performed calculations on lithium in contact with PCL:LiTFSI, and showed that LiTFSI can decompose into, among other compounds, an NSC compound (resulting from breaking the S–O bond from an NSO_2_C fragment).^37^ Since the binding energy (BE) of NSC in the S 2p spectrum fits with that for the previously undefined peak, we find it likely that this NSC compound is the origin of this peak.^37^ An NSC compound would also give contributions to the N 1s and C 1s spectra. In the N 1s spectra, this contribution is expected to overlap with the peak previously assigned to Li_3_N. Based on the results from Wu et al., and the observation in the S 2p spectra, it is likely that the N 1s peak partially originates from the NSC. It is, however, deemed possible that a dominating part of the N 1s peak originates from Li_3_N, due to the abundance of lithium, which can drive the decomposition of the salt to the end. Also, the lack of a clear NSC peak in the C 1s spectra, predicted to be significantly lower in BE compared to the hydrocarbons of PTMC, supports this conclusion.^37^

### LLZO:Polymer Interface: Surface Concentrations in the Pristine CPEs

The interfaces formed between the polymer matrix and ceramic particles are crucial for ionic transport in CPEs. These can provide pathways for Li-ion conduction, resulting in improved overall ionic conductivity in CPEs as compared to SPEs. However, the efficiency of interfacial transport is strongly dependent on surface chemistry, where it appears that both advantageous and disadvantageous surfaces exist, which promote or retard ionic mobility, respectively. By investigating the surface of the pristine CPEs, which contain particles embedded in the polymer, information on the ceramic-polymer interfacial chemistry can be obtained. The resulting polymer-ceramic interfaces will also be dependent on the filler concentration.

**Figure 5:**
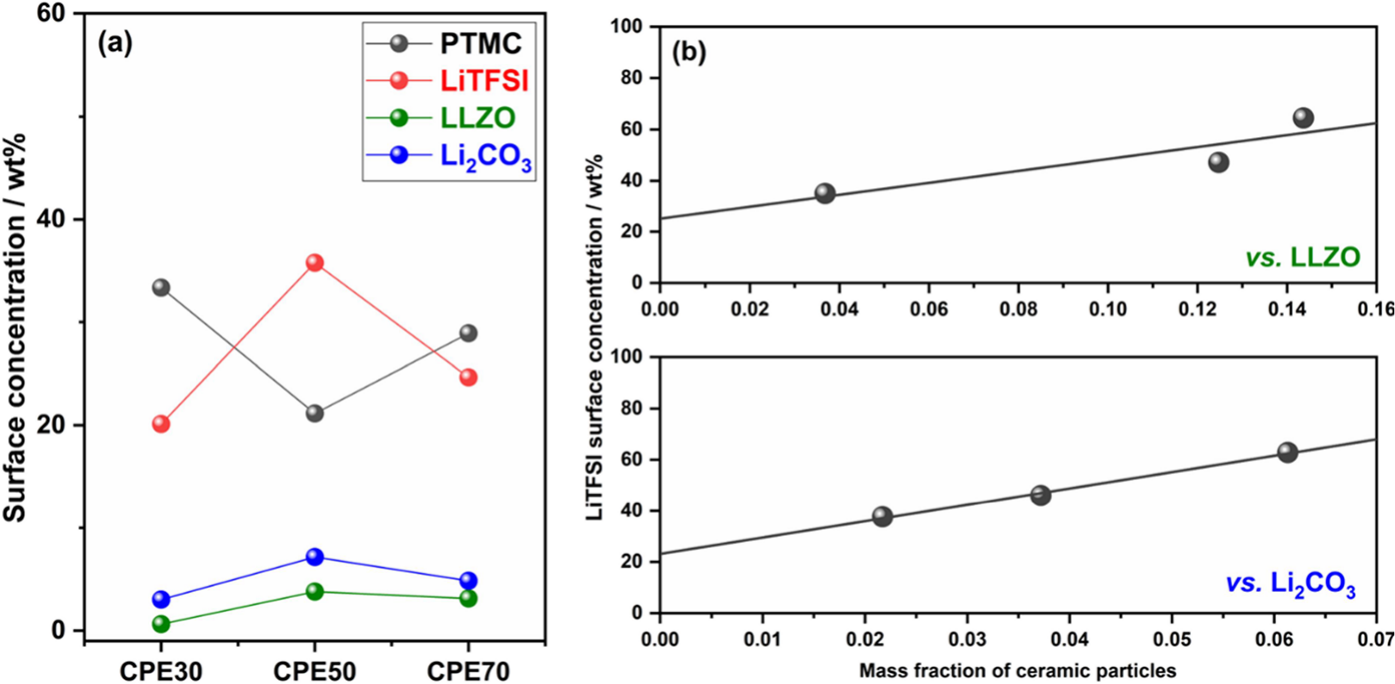
(a) Concentration of CPE components in the surface region before Li deposition calculated from O 1s data. (b) Concentration of LiTFSI vs concentration of LLZO and Li_2_CO_3_ in the surface region, calculated from the O 1s and C 1s data, respectively. The lines in (b) represent linear fits to the data.

From the O 1s spectra, it can be noted that the intensity ratio between LLZO and LiTFSI is roughly the same for CPE50 and CPE70 (0.09–0.10). This is unexpected, considering that the total concentration of LiTFSI in the sample should be lower the higher the LLZO loading is. Furthermore, from the C 1s spectra, it is noted that the intensity ratio between LiTFSI and PTMC is not constant. To evaluate these observed trends further, the surface concentrations of PTMC, LiTFSI, LLZO, and Li_2_CO_3_ were calculated using the peak intensity values in Table S2, the eqs S1 and S2 in SI, and plotted in Figure 5a.

From Figure 5a, it is clear that the surface concentrations of LLZO obtained from O 1s do not follow the bulk concentration of LLZO in the CPEs, but the concentration is instead highest for CPE50, followed by CPE70, with CPE30 having the lowest surface concentration. This trend is further verified by the observation of the same trend in the Li_2_CO_3_ surface concentration as seen from the C 1s spectra. Li_2_CO_3_ is usually found on the surface of LLZO particles. Since there should be no other sources of Li_2_CO_3_ in the pristine CPEs, Li_2_CO_3_ serves as a second indicator for the LLZO surface, assuming a constant surface concentration of Li_2_CO_3_ on these surfaces.^36,38,39^ The surface concentration of LLZO is lower at 70 wt % than at 50 wt %, suggesting that there could be severe agglomeration at this high particle loading and that an enrichment of LLZO instead occurs in the CPE70 bulk. At the same time, since the LLZO peaks in the O 1s spectra before deposition are quite small, it should be noted that there could be significant uncertainty in the surface concentration values.

Interestingly, the same trend with the highest values for CPE50, which is observed for LLZO and Li_2_CO_3_, is also observed for LiTFSI. As salt accumulation on the SPE surface has not been observed to be pronounced for PTMC:LiTFSI (i.e., without LLZO),^28^ this trend indicates that the enrichment of LiTFSI at the surface of the samples is triggered by the addition of LLZO particles to the polymer electrolyte matrix. Previously, it has been experimentally demonstrated through Fourier-transform infrared spectroscopy (FTIR) that there could be an interaction between TFSI anions and the surface of LLZO particles through Lewis acid–base interactions, resulting in TFSI enrichment at the surface.^13,14,40−45^ However, this effect could be significantly limited when Li_2_CO_3_ is covering the surface of the LLZO particles. Still, these PES results clearly indicate an accumulation of TFSI anions at the surface of the LLZO particles. A similar effect of salt enrichment on surfaces has been suggested for other CPEs before in both experimental data and from computational studies.^46,47^

When the surface concentration of LiTFSI is plotted against the surface concentration of Li_2_CO_3_ in Figure 5b, a linear trend is displayed based on the three data points at 30, 50, and 70 wt % of LLZO. When extrapolated to 0 wt % LLZO (or Li_2_CO_3_), a LiTFSI concentration of ∼23 (or ∼25 wt %) is observed, which is close to the expected bulk salt concentration (28 wt %) in the polymer matrix (i.e., truly at 0 wt % LLZO). This corroborates the accumulation of LiTFSI at the particle surface, irrespective of whether LLZO is partially covered by Li_2_CO_3_.

### Reactions after Li Deposition: PTMC Decomposition

Considering the intrinsic reactivity of the Li-metal electrode, the chemical and electrochemical stabilities of any employed electrolyte are decisive for electrochemical performance. The interfacial chemistry of solid-state electrolytes is, as has been shown, determining many of the resulting battery properties.^48^ As compared to neat SPEs, the surface chemistry of the LLZO particles, as investigated above, could also influence the properties of the CPE|Li interfaces.

**Figure 6:**
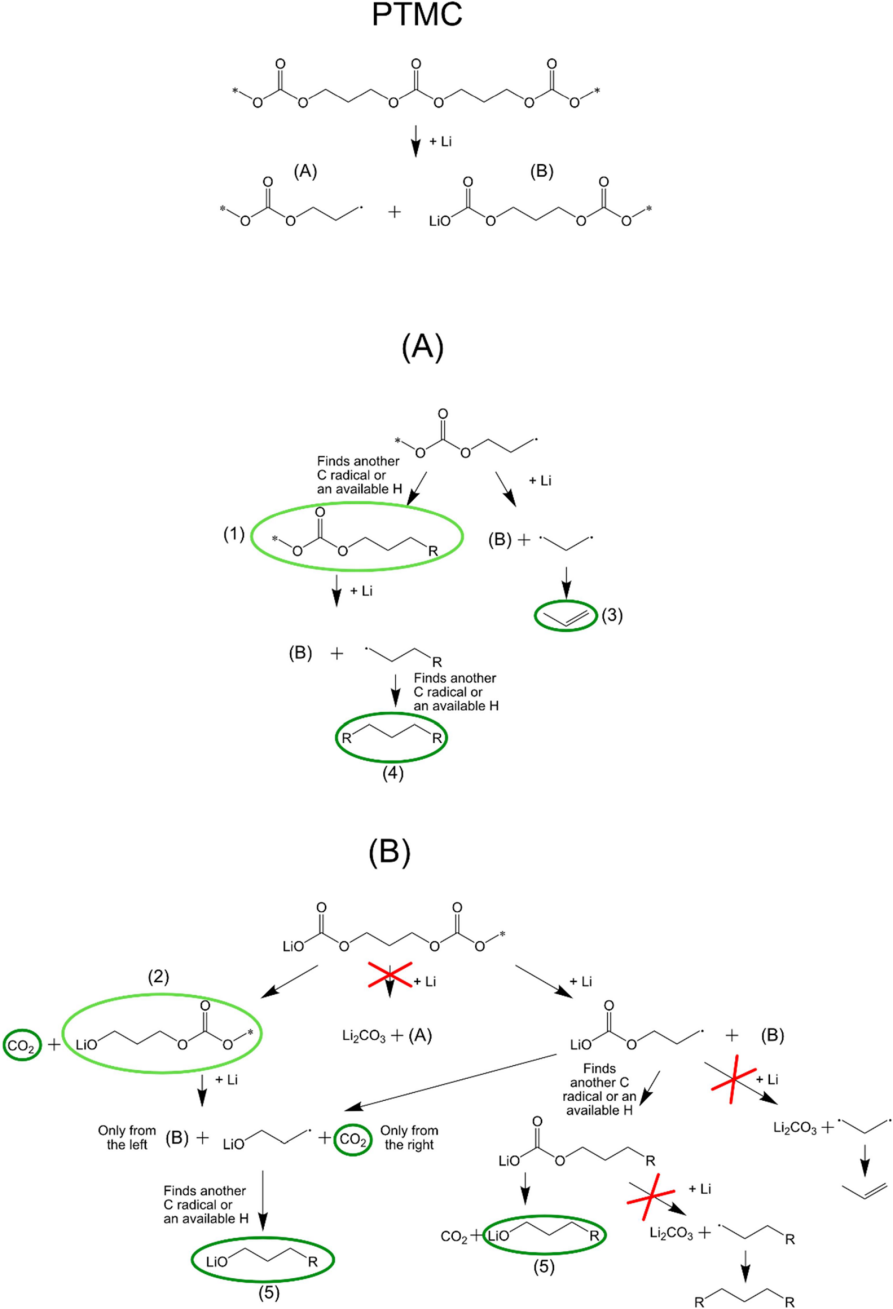
Suggested decomposition pathways for the PTMC polymer. A and B represent two possibilities for polymer decomposition after the first Li attack.

After Li deposition, the CPEs can decompose to form a range of organic and inorganic breakdown products. From a polymer perspective, it was previously suggested for the polymer PEO that an increase in hydrocarbons could be related to the formation of polyethylene.^30^ PTMC, on the other hand, has a three-carbon fragment in the main chain, and degradation to form ethylene is therefore not likely to occur, which is why the increase in hydrocarbons observed in the PTMC:LiTFSI system was left unsolved in our previous study.^28^ However, a closer look at the PTMC decomposition pathways gives insights into this matter and can explain the increase in the level of hydrocarbons, which is also observed in the current PES measurements. The possible decomposition pathways, based on Li cleaving the C–O bond outside the carbonate unit (found to be the most energetically favorable by the calculations of Ebadi et al.^49^) are given in Figure 6. The argument for the cleavage primarily starting in the main chain, rather than at the end groups, is based on the low concentration of end groups at the surface for Li to react within the high-*M*_w_ systems employed, as well as the absence of Li metal and a large amount of decomposition species. Some observations in the PES spectra and assumptions based on previous studies^28,37^ can narrow down the decomposition pathways slightly and allow us to find the possible components of the decomposed PTMC.

First, the decrease of carbonate species in the C 1s spectra indicates that any steps that form Li_2_CO_3_ are unlikely. While Li_2_CO_3_ formed from PTMC could in principle decompose further (with the addition of 2 Li) into Li_2_O and CO, no CO was observed in an online electrochemical mass spectrometry (OEMS) study by Sångeland et al.^50^ Therefore, the formation of Li_2_CO_3_ from PTMC is here assumed to be insignificant. The steps leading to Li_2_CO_3_ have therefore been crossed out.

Second, the release of large amounts of CO_2_ seen in the study of Sångeland et al. suggests that the PTMC carbonate ending with a Li will not be long-lived but will instead form alkoxides and CO_2_ rather quickly. Thereby, products with PTMC carbonate ending with a Li can be excluded from the PES spectra.

Third, radicals are generally not long-lived, and products with radicals are therefore not thought to be part of the PES spectra after deposition.

Fourth and finally, it is likely that stable intermediate products, where not all C–O bonds outside the carbonyl unit have yet been broken, still exist in the surface region. This is motivated by the fact that pristine PTMC can still be observed after lithium deposition, thus indicating incomplete decomposition of the polymer components.

Based on these four points, some possible decomposition products can be suggested, as illustrated in Figure 6. The “main” products are marked with dark green circles, and intermediate products are marked with light green. These possible decomposition products include PE-like R-R’ chains, linked to either H or even more hydrocarbon units, which would show up as hydrocarbon in the C 1s spectra. These species includes propylene, which through a Li-triggered polymerization similar to that of ethylene in our previous PEO study,^30^ could form polypropylene. Computational studies could possibly shed further light on these issues, as has been shown in previous studies on similar systems.^49,51^

Finally, the PTMC chain ending with the R would also contribute to the hydrocarbon peak. Other possible decomposition products are Li alkoxide attached to a poly(ethylene) PE-like chain or the regular PTMC chain, and CO_2_ that would leave the sample as a gas. Li alkoxide is indeed also observed in the PES spectra after Li deposition.

In addition to the decomposition pathways presented in Figure 6, Li_2_O is observed in the PES spectra, in line with previous studies on PTMC-based electrolytes.^28,52^ Several potential options for the formation of Li_2_O can be identified. First, Li_2_O could at least partially come from the decomposition of the Li alkoxides. In this context, it is important to note that lithium-induced decomposition is most severe for CPE50. However, in the O 1s spectra, the ratio of intact PTMC to Li alkoxide is smaller for CPE50 than for CPE30, while the Li_2_O peak is still larger in the case of CPE50. The Li_2_O peak is expected to grow at the cost of Li alkoxide, and with a higher decomposition of the Li alkoxide in CPE50 compared to CPE30, this can explain this disruptive trend in the O 1s data. Second, there is also the possibility that the Li_2_O formed is from the reaction between Li and Li_2_CO_3_ or LiOH on the LLZO surface. Apart from Li_2_O, Li_2_CO_3_ would in this case form CO (through reactions involving two extra Li), and LiOH would form 1/2 H_2_ (while adding one Li). Third, the observed Li_2_O could also come from a reaction between Li and LLZO itself, i.e., directly dependent on the ceramic CPE component itself. However, this is deemed unlikely, since the LLZO peak in the O 1s spectra actually increases after deposition. Fourth, Li_2_O is expected from the decomposition of the TFSI anion,^37^ but considering the amount of Li_2_O formed, this would require substantial TFSI accumulation and decomposition. It is possible that a combination of the different non-PTMC sources would be enough to explain the Li_2_O peak.

On a final note, the increase of the LLZO observed after lithium deposition can be explained by the formation of gaseous species from the material that initially covers the LLZO particles, e.g., Li_2_CO_3_. This gives further strength to the hypothesis that PTMC decomposes into CO_2_, and Li_2_CO_3_ decomposes into CO. It is, however, not possible to rule out LLZO decomposition and generally difficult to specifically identify any of the potentially resulting products, since no La or Zr spectra were measured.

## Conclusions

In this work, the surface characteristics of composite polymer electrolytes with a PTMC:LiTFSI polymer matrix and with LLZO as a ceramic ion-conductive filler have been investigated, analyzing both the interaction between the polymer matrix and the ceramic particles and the formed interface between the CPE and Li metal. The results reveal that the surface concentration of LiTFSI is linearly increasing with increasing LLZO ceramic concentration at the surface. This suggests an accumulation of LiTFSI salt species near the partially Li_2_CO_3_-covered LLZO particle surfaces. The presence of Li_2_CO_3_ has previously been shown to retard the ionic transport in PTMC:LLZO CPEs. Furthermore, based on the spectra acquired after Li metal deposition, reaction pathways are suggested in which the decomposition of PTMC results in PE-like chains and polypropylene as major decomposition products. In addition, TFSI breaks down into its typical inorganic byproducts. Notably, the degradation of both polymer and salt is particularly pronounced for the CPE with 50 wt % of LLZO, at which also the highest surface concentration of Li_2_CO_3_-covered particles is observed. These partially Li_2_CO_3_-covered LLZO particles can possibly undergo partial decomposition, resulting in the formation of Li_2_O and CO. This study sheds light on the need to better explore the surface and interfacial properties of CPEs, for a better understanding of the influence of the ceramic filler’s surface chemistry on ionic transport. This will be necessary to tailor these CPEs for functional use in solid-state Li-metal battery applications.

## Supporting Information Available

Surface concentration of different species in CPEs; S 2p and N 1s core-level spectra of all CPEs before and after Li deposition; and binding energy values and their corresponding intensities for C 1s and O 1s core levels before and after Li deposition for all CPEs (PDF)
