# Supplementary material for: Interfacial Structure and Reactions in Li6.7Al0.3La3Zr2O12-Doped Polycarbonate-Based Composite Polymer Electrolytes
Source: ACS Appl Polym Mater. 2025 Feb 28;7(5):3112–21. doi: 10.1021/acsapm.4c03865 (PMC11915194; doi:10.1021/acsapm.4c03865)
Supplement: Supplementary file 1 — ap4c03865_si_001.pdf [file ap4c03865_si_001.pdf]

Supporting Information

Interfacial Structure and Reactions in $Li_{6.7}Al_{0.3}La_3Zr_2O_{12}$-Doped Polycarbonate-Based Composite Polymer Electrolytes

*Kenza Elbouazzaoui[1], Edvin K.W. Andersson[1], Yi-Chen Weng[2], Daniel Friesen[1], Kristina Edström[1], Erika Giangrisostomi[3], Ruslan Ovsyannikov[3], Daniel Brandell[1, *], Jonas Mindemark[1], Maria Hahlin[1,2]*

[1] *Department of Chemistry – Ångström Laboratory, Uppsala University, Box 538, SE-751 21, Uppsala, Sweden*

[2] *Department of Physics and Astronomy, Uppsala University, Box 516, Uppsala, Sweden*

[3] *Institute for Methods and Instrumentation for Synchrotron Radiation Research, Helmholtz-Zentrum Berlin für Materialien und Energie, Albert-Einstein-Str. 15, 12489 Berlin, Germany*

* Corresponding Author: daniel.brandell@kemi.uu.se

Table of contents: Page:

1. Calculations of concentrations based on XPS data S2
2. Additional XPS data S3
3. Binding energies S4

1. Calculations of concentrations based on XPS data

The surface concentration in the polymer matrix of the samples were calculated from the intensity of the peaks in the C 1s spectra. What follows is a description of the calculations.

The atomic concentration of a species corresponds to the intensity of the peak of that species when comparing with the total intensity of all peaks within a core level:

$$C_{\mathrm{A}} = \frac{I_{\mathrm{A}}}{\sum I_i} \tag{S1}$$

By multiplying the intensity of each species with their respective molecular weight the mass fraction is obtained:

$$m_{\mathrm{A}} = \frac{I_{\mathrm{A}} \times M_{\mathrm{A}}}{\sum (I_i \times M_i)} \tag{S2}$$

The calculated mass fraction represents surface concentration since the PES originates mainly from the top surface. Plus, since only carbon is considered and all species with carbon are assumed to either be outside of the LLZO bulk, the surface concentration is assumed to be the surface concentration in the polymer matrix.

2. **Additional XPS data**

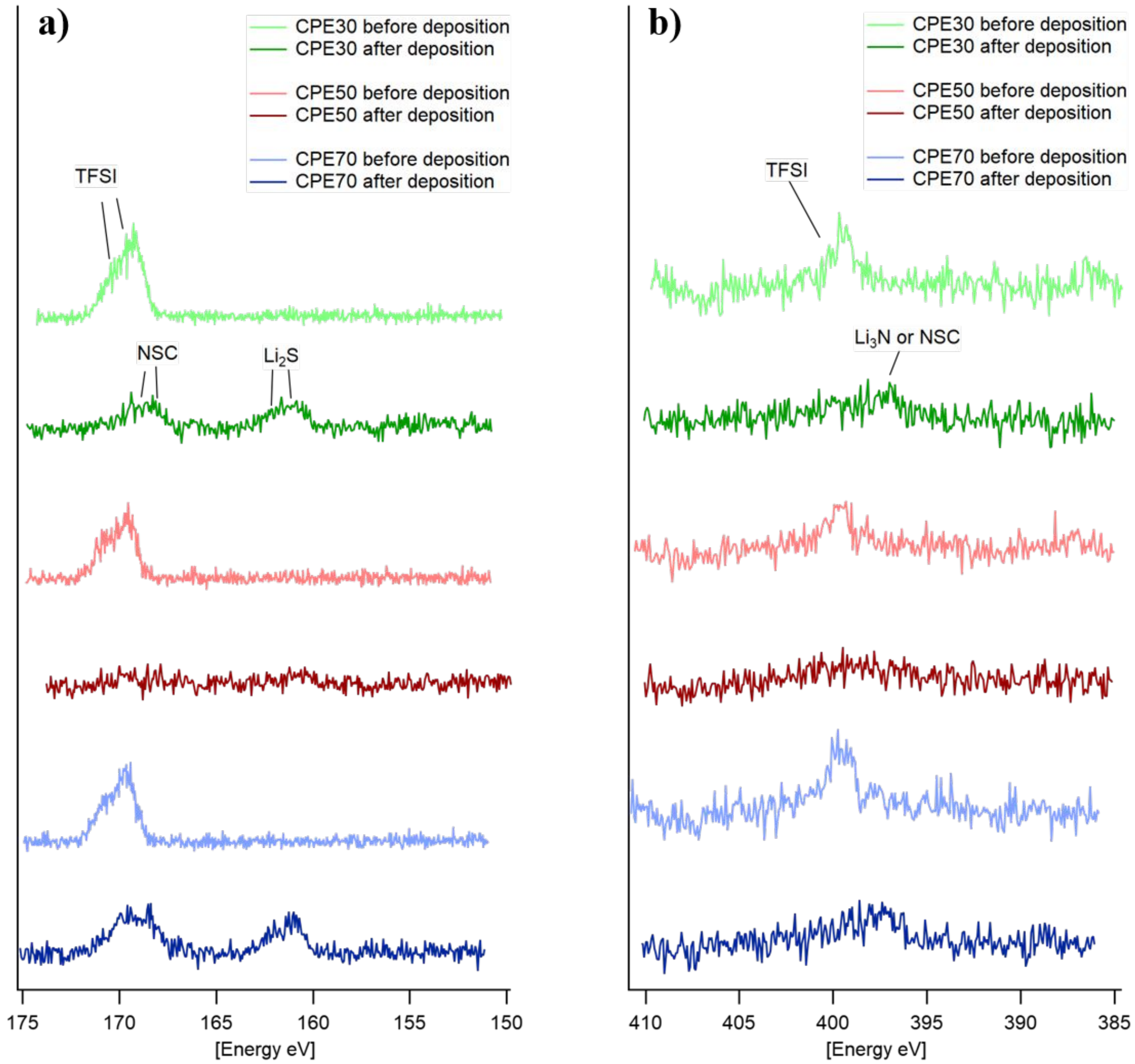

Figure S1. (a) S 2p, (b) N 1s core level spectra of all CPEs before and after Li deposition.

3. Binding energies

Table S1. Binding energy values and their corresponding intensities for the C 1s core level before and after Li deposition for all CPEs.

	C 1s					
	**Before Li deposition**			**After Li deposition**		
	Peak	BE (eV)	Int (a.u.)	Peak	BE (eV)	Int (a.u.)
**CPE30**	PTMC1	290.8693012	8189.204	PTMC1	290.8426295	2955.264
	PTMC2	286.9693012	16376.55	PTMC2	286.9426295	6506.018
	PTMC3	285.6693012	8189.154	LiTFSI	292.9726295	2330.821
	LiTFSI	292.9984642	3515.375	Total Hydrocabon	285.5176635	44465.1
	Hydrocarbon	284.9527667	21901.34	Li_2CO_3	289.8426295	1799.001
	Li_2CO_3	289.8693012	1027.756	U1	288.3926295	2118.246
	U1	288.4250102	2295.506			
**CPE50**	PTMC1	290.8147505	5124.527	PTMC1	290.1334943	1395.793
	PTMC2	287.2147505	10251	PTMC2	286.5334943	2148.839
	PTMC3	285.6147505	5125.476	LiTFSI	292.2634943	708.8886
	LiTFSI	293.0024407	6158.89	Total Hydrocabon	285.1680103	23940.6
	Hydrocarbon	285.4073749	14994.44	Li_2CO_3	289.1334943	430.8423
	Li_2CO_3	289.8147505	2395.415	U1	287.6834943	491.1989
	U1	288.5949297	1333.769			

CPE70	PTMC1	290.5684094	7086.798	PTMC1	291.0184808	3449.439
	PTMC2	286.8684094	14176.52	PTMC2	287.3184808	6120.698
	PTMC3	285.3684094	7088.21	LiTFSI	293.1484808	2664.539
	LiTFSI	293.0031581	4292.094	Total Hydrocabon	285.5471384	35042.25
	Hydrocarbon	285.0456276	17995.38	Li_2CO_3	290.0184808	2455.3
	Li_2CO_3	289.5684094	1641.718	U1	288.5684808	1137.572
	U1	288.3734545	1756.563			

Table S2. Binding energy values and their corresponding intensities for the O 1s core level before and after Li deposition for all CPEs.

	O 1s					
	**Before Li deposition**			**After Li deposition**		
	Peak	BE (eV)	Int (a.u.)	Peak	BE (eV)	Int (a.u.)
CPE30	PTMC1	533.6763105	12652.63	PTMC1	533.9814785	3010.037328
	PTMC2	532.2763105	6327.223	PTMC2	532.5814785	1245.680297
	LiTFSI	532.1577867	5427.907	LiTFSI	532.5794785	1278.373093
	Li_2CO_3	532.3798783	2378.729	Li_2CO_3	532.8354785	688.0249954
	LiOH	531.4798783	7666.169	LiOH	531.9354785	7998.044876
	LLZO	529.3798783	176.0616	LLZO	529.8354785	1127.911742
				LiRO	530.5896174	32234.93465
				Li_2O	528.2184278	2289.15377
CPE50	PTMC1	533.2998581	5670.971	PTMC1	533.5337799	1719.787075
	PTMC2	531.8998581	2835.103	PTMC2	532.1337799	779.2575532
	LiTFSI	531.8978581	6815.841	LiTFSI	532.1317799	1420.910191
	Li_2CO_3	532.1538581	3975.355	Li_2CO_3	532.3877799	601.0170661
	LiOH	531.2538581	6261.999	LiOH	531.4877799	3367.740443
	LLZO	529.1538581	742.1373	LLZO	529.2877799	1881.045113

				LiRO	530.2607131	15907.86016
				Li_2O	527.5674247	4387.93432
**CPE70**	PTMC1	533.5010163	16151.69	PTMC1	534.0562275	4149.820372
	PTMC2	532.1010163	8077.616	PTMC2	532.6562275	1830.280009
	LiTFSI	532.0990163	9789.818	LiTFSI	532.6542275	2721.112045
	Li_2CO_3	532.3550163	5604.985	Li_2CO_3	532.9102275	883.4898658
	LiOH	531.4550163	11317.29	LiOH	531.8102275	14126.08951
	LLZO	529.3550163	1275.935	LLZO	529.8102275	3286.229515
				LiRO	530.5952219	19617.69809
				Li_2O	528.0056347	2846.219577
